# A MODIFIED MULTIFACTORIAL INTERVENTION FOR OLDER ADULTS WITH DIFFERENT KNEE OSTEOARTHRITIS SEVERITY

**DOI:** 10.1093/geroni/igae098.3004

**Published:** 2024-12-31

**Authors:** Suparb Aree-Ue, Inthira Roopsawang, Natthayawadee Saraboon

**Affiliations:** Mahidol University, Bangkok, Krung Thep, Thailand; Mahidol University, Bangkok, Krung Thep, Thailand; Boromarajonani College of Nursing, Nakhon Phanom, Nakhon Phanom, Nakhon Phanom, Thailand

## Abstract

Knee osteoarthritis (OA) is a common health condition affecting older adults’ health worldwide. Several modalities to mitigate disease severity have been implemented; however, whether those interventions benefit all stages of disease severity has not been explored. This quasi-experimental study aimed to compare the effect of a modified multifactorial intervention program on health outcomes between older adults with mild and moderate knee OA severity. Ninety prospective participants who met the inclusion criteria were assigned into Group 1 (mild symptoms) or Group 2 (moderate symptoms). The modified multifactorial intervention program—health education about knee OA, weight control, quadriceps exercise, and home visits—was delivered to both groups lasting 12 weeks. The health outcomes (weight, psychological well-being, muscle strength, and knee OA severity) were assessed at baseline, 8-, and 12-week after enrollment using standard self-reported questionnaires and a 30-second sit-to-stand test. Data were analyzed using descriptive, independent-t-test, MANCOVA, and repeated measure MANOVA. Results revealed that the remaining 85 participants’ age ranged from 60 to 85 years, with a mean age of 68.8 years (SD = 8.6 years); almost were females (95.2%; n = 79). Although knee OA severity and psychological well-being significantly differed between the groups at baseline, there was no interaction effect on other dependent variables. Weight and muscle strength evaluated by a 30-second sit-to-stand test has no significant difference between groups. Results can be concluded that the modified multifactorial intervention program is beneficial to promote better knee joint function and health among older people with mild and moderate knee OA.

